# Cyp17a2 is involved in testicular development and fertility in male Nile tilapia, *Oreochromis niloticus*

**DOI:** 10.3389/fendo.2022.1074921

**Published:** 2022-11-29

**Authors:** Lanying Yang, You Wu, Yun Su, Xuefeng Zhang, Tapas Chakraborty, Deshou Wang, Linyan Zhou

**Affiliations:** ^1^ Key Laboratory of Freshwater Fish Reproduction and Development, Ministry of Education, Southwest University, Chongqing, China; Fisheries Engineering Institute, Chinese Academy of Fishery Sciences, Beijing, China; College of Fisheries, Southwest University, Chongqing, China; ^2^ Faculty of Agriculture, Kyushu university, Fukuoka, Japan

**Keywords:** Nile tilapia, Cyp17a2, DHP, cortisol, spermatogenesis, male fertility

## Abstract

**Background:**

Steroid hormones play an essential role in many reproductive processes of vertebrates. Previous studies revealed that teleost-specific Cyp17a2 (cytochrome P450 family 17 subfamily a 2) might be required for the production of cortisol in the head-kidney and 17α,20β-dihydroxy-4-pregnen-3-one (DHP) in ovary during oocyte maturation. However, the role of Cyp17a2 in male reproduction remains to be largely unknown. The aim of this study was to explore the essentiality of cyp17a2 gene in male steroidogenesis, spermatogenesis, and male fertility.

**Methods:**

A homozygous mutation line of cyp17a2 gene was constructed in tilapia by CRISPR/Cas9 gene editing technology. The expression level of germ cell and meiosis-related genes and steroidogenic enzymes were detected by qRT-PCR, IHC, and Western blotting. EIA and LC-MS/MS assays were used to measure the steroid production levels. And sperm quality was examined by Sperm Quality Analyzer software.

**Results:**

In this study, cyp17a2 gene mutation resulted in the significant decline of serum DHP and cortisol levels. On the contrary, significant increases in intermediate products of cortisol and DHP were found in cyp17a2-/- male fish. The deficiency of cyp17a2 led to the arrest of meiotic initiation in male fish revealing as the reduction of the expression of germ cell-related genes (vasa, piwil, oct4) and meiosis-related genes (spo11 and sycp3) by 90 dah. Afterwards, spermatogenesis was gradually recovered with the development of testis in cyp17a2-/- males, but it showed a lower sperm motility and reduced fertility compared to cyp17a2+/+ XY fish. Deletion of cyp17a2 led to the abnormal upregulation of steroidogenic enzymes for cortisol production in the head-kidney. Moreover, unaltered serum androgens and estrogens, as well as unchanged related steroidogenic enzymes were found in the testis of cyp17a2-/- male fish.

**Conclusion:**

This study proved that, for the fist time, Cyp17a2 is indispensable for cortisol and DHP production, and cyp17a2 deficiency associated curtailed meiotic initiation and subfertility suggesting the essentiality of DHP and cortisol in the male fertility of fish.

## Introduction

It is well known that steroid hormones play essential roles in gonadal development, gametogenesis, and fertility in vertebrates (1–5). In fish, 17α, 20β-dihydroxy-4-pregnen-3-one (17α, 20β-DP, DHP) and 17α, 20β, 21-trihydroxy-4-pregnen-3-one (17α, 20β, 21-P) are the two main maturation-inducing hormones (6, 7). Previous studies suggested that DHP might be involved in meiotic initiation and spermiation (8–11). Cortisol, the primary glucocorticoid in fish, regulates stress, immune response, energetic metabolism and osmoregulation, and recently being thought to be involved in testicular development and spermatogenesis in male fish (12–17).

In vertebrates, cytochrome P450s (17α-hydroxylase/C17–20 lyase, Cyp17) occupied the center of biosynthesis of steroid hormones. In mammals, Cyp17a had been proved to be required for the production of androgens and estrogens in gonadal tissues, and glucocorticoid in adrenal tissues (18, 19). In contrast, duplicated *cyp17a* genes, i.e. *cyp17a1* and *cyp17a2*, were isolated from the genomes of several teleosts and suspected to synergistically catalyzing the synthesis of C18, C19 and C21 steroid hormones (20–22). Previous reports revealed that *cyp17a1* gene mutation ceased both androgens and estrogens production, and caused spermiation disorder, infertility and all-male population production (23–25). Cyp17a2, a teleost-specific Cyp17a steroidogenic enzyme, possessed different tissue expression patterns and different steroidogenic activities from Cyp17a1 (20). Previous studies indicated that Cyp17a2 might be required for the biosynthesis of DHP during oocyte maturation as well as glucocorticoid production in the head-kidney (20, 26). However, the functions of Cyp17a2 in fish steroidogenesis and reproduction are still vague and require detailed investigation.

Nile tilapia (*Oreochromis niloticus*), a commercially important aquaculture fish across globe, is an ideal model to study the molecular mechanisms on fish steroidogenesis due to the well-established gene editing technology, availability of monosex fish and a short spawning cycle (14-day) (27, 28). Using tilapia, earlier we reported that Cyp17a2 was abundantly expressed in ovarian follicular cells, testicular interstitial cells and interrenal cells of head-kidney and possessed unique hydroxylase activity (20). In our present study, a null mutation line of *cyp17a2* gene was constructed by CRISPR/Cas9 technology to assess the functional implication of Cyp17a2 in teleostean steroidogenesis and gonadal maintenance. Comparative analyses were conducted to assess the effects of *cyp17a2* gene mutation on DHP and cortisol production, meiotic initiation, spermatogenesis, sperm health and fertility. Taken together, our findings provide important insights into the molecular mechanisms and endocrine regulation of male fish fertility.

## Materials and methods

### Animals

In this study, Nile tilapia (*Oreochromis niloticus*) were acclimatized in recirculating aerated freshwater tanks at 26 °C under a natural photoperiod before use. All animal experiments were conducted per the regulations of the Guide for Care and Use of Laboratory Animals approved by the Committee of Laboratory Animal Experimentation of Southwest University, China.

### Production and characterization of Cyp17a2 polyclonal antibody

The recombinant construct of Cyp17a2 was prepared by cloning the 1566 bp *cyp17a2* ORF sequences into a pCold I expression vector. The recombinant plasmid with His-tag at its N-terminal was transformed into *Escherichia coli*, and expressed with the induction of isopropyl β-D-l-thiogalactopyanoside (IPTG, 1 mmol/L). The His-Cyp17a2 recombinant proteins (25-30 µg) was purified with a Ni-NTA super flow cartridge (Qiagen, Hilden, Germany) and used as an antigen to immunize female rabbits three times at 15-day intervals. Ten days after the last immunization, rabbit serum was collected and purified by affinity chromatography on Sepharose 4B Fast Flow resin (Sigma, Darmstadt, Germany) combined with the Cyp17a2 recombinant protein. To confirm the specificity of the polyclonal antibody, total proteins extracted from testis, ovary, head-kidney, brain, and muscle from 180 dah tilapia were separated using 12.5% SDS-PAGE under reducing conditions. Western blotting was performed according to the methods described previously (29) and the antibody against tilapia Cyp17a2 was diluted at 1:1000.

### Cellular localization of Cyp17a2 in gonads and head-kidney of tilapia by Immunohistochemistry

To determine the cellular localization of Cyp17a2 in the testis and head-kidney of tilapia, the testes and head-kidneys from wild-type males at different development stages were dissected and fixed in Bouin’s solution for 12 hours at room temperature, then dehydrated and embedded in paraffin. The tissue blocks were then sliced into 5-μm-thickness sections, then the sections were treated in a blocking solution (5% BSA diluted in 1x PBS) (Sangon Biotech, China), incubated with the primary antibody against Cyp17a2 overnight at 4°C and rinsed with 1x PBS five times for 5 min per wash. In this study, the anti-Cyp17a2 polyclonal antibody was diluted at 1:1000 before use. As a negative control, the primary antibody was replaced with normal rabbit serum. The slides were then incubated with anti-rabbit immunoglobulin G (diluted at 1:1000) at room temperature for 1 h, and then rinsed with 1x PBS three times for 5 min per wash. Immunoreactive signals were visualized using diaminobenzidine tetrachloride (DAB) (Sigma, Germany) as the substrate. Sections were then counterstained with hematoxylin. Finally, all the images for these sections were acquired with an Olympus BX5 light microscope (Olympus, Japan).

### Knockout of the *cyp17a2* gene by CRISPR/Cas9

A homozygous mutation line of *cyp17a2* was constructed in tilapia to elucidate the functions of Cyp17a2 in fish steroidogenesis, spermatogenesis, and male fertility by using CRISPR/Cas9 technology. The guide RNA (gRNA) target site was selected from sequences corresponding to GGN18NGG on the sense strand of DNA (http://zifit.partners.org/ZiFiT/). The synthesis of gRNA and Cas9 mRNA, and the screening and establishment of homozygous mutation line were carried out according to previous report (25).

### Histological observation and change of gene expression

Testes at different developmental stages (75, 90 and 180 dah) and head-kidneys (180 dah) from wild-type and *cyp17a2* ^-/-^ fish were dissected and embedded in paraffin. IHC analysis was conducted as described above. Anti-Vasa (germ cells marker, diluted at 1:1000), -PCNA (Proliferating cell nuclear antigen, diluted at 1:500, Cusabio, Wuhan, China), -StAR1 (Steroidogenic acute regulatory protein 1, diluted at 1:500), -Cyp17a1 (cytochrome P450 family 17 subfamily a 1, Leydig cell marker, diluted at 1:1000), -Cyp11b2 (Cytochrome P450, family 11, subfamily B, polypeptide 2, Leydig cell marker, diluted at 1:1000), and -Cyp17a2 polyclonal antibodies were used to assess the impacts of *cyp17a2* mutation on spermatogenesis and head-kidney development.

### Quantitative real-time PCR

Gonads and head-kidneys were collected from wild-type and *cyp17a2* ^-/-^ XY fish (n≥3 fish/genotype). Total RNA was isolated for each sample using RNAiso Plus (Takara, Dalian, China), and reverse transcribed using the Prime Script RT Master Mix Perfect Real Time Kit according to the manufacturer’s instructions (Takara, Dalian, China). All qRT-PCRs were carried out in an ABI-7500 fast Real-time PCR machine (Applied Biosystems, USA), and all experiments were performed according to the manufacturer’s instructions. The relative abundance of target genes was normalized to *β-actin* using R=2^-ΔΔCt^ formula (30). The primer sequences used for the PCR reactions were listed in **Table 1**.

**Table 1:** Primers used in the present study.

primer name	primer sequence (5'-3')	accession number	purpose
*cyp17a2*-Cas9-F	GGCAGTCTCCCCTGGCTTGG	NM_001279458	gRNA amplification
*cyp17a2*-Cas9-R	TGTAAACATTTATGACAGTGGG		
*cyp17a2*-T-F	TCGTTCCTGCCTCCTTCG		Sequencing and mutant screening
*cyp17a2*-T-R	TGGGCTGATGAACATCACTCCT		
*cyp17a2*-P-F	CTGAAACTGAACCCTCGGCT		
*cyp17a2*-P-R	CTGTGGGACATCTGCGTGAA		
*cyp17a1*-Q-F	TGTCATCAACCAGCATGTGCAC	NM_001279765	qRT-PCR
*cyp17a1*-Q-R	ACTTCCACGTAGCACTGTAGTC		
*StAR1*-Q-F	CTGAAACTGTTGCTGCGAATGGA	XM_003445605	
*StAR1* -Q-R	GGTCTCTGCGGATACCTCGTG		
*cyp11b2*-Q-F	AAAGAAGTCCTCAGGTTGTACC	XM_003450906	
*cyp11b2*-Q-R	GACCAAAGTTCCAGCAGGTATG		
*cyp21a2-*Q-F	AATGCGGAACAACAACTATGG	XM_019365158	
*cyp21a2-*Q-R	TATGTTGCAACCTCCCCCAG		
*vasa-*Q-F	GGGAGCTGATCAACCAGATT	XM_019351278	
*vasa-*Q-R	CTGGTGTTCCACACAACACA		
*piwil-*Q-F	ACATCCCACAGCACAAGTTGAC	XM_003445546	
*piwil-*Q-R	CTGCCTCAAGCTGACCATAAAG		
*cyp26b1-*Q-F	CTTGCCCTTTCCCGTTGC	XM_005471225	
*cyp26b1-*Q-R	GCGGTTCCCGAAGAGGTGT		
*spo11*-Q-F	CGAGAAGGATGCGACGTTCCAGAG	XM_013274323	
*spo11*-Q-R	GAGCGTCCTTGGGAACCCGC		
*sycp3*-Q-F:	CTGACTTTGAGGAGGAGGCG	XM_003439369	
*sycp3*-Q-R:	GCTTGTGTTGGCTTCCCTTC		
*β-actin-*Q-F	GGCATCACACCTTCTACAACGA	XM_003443127	
*β-actin*-Q-R	ACGCTCTGTCAGGATCTTCA		

F, Forward primer; R, Reverse primer, respectively. T, test; P, PAGE; Q, qRT-PCR.

### Western blotting

Western blotting was performed to detect the expression of steroidogenic enzymes in the testes of *cyp17a2* ^-/-^ XY tilapia. Total proteins were extracted from the testes of *cyp17a2* ^+/+^ XY (n=3) and *cyp17a2* ^-/-^ XY (n=3) fish at 180 dah, diluted to a final concentration of 20 mg/mL and stored at -20 °C until use. Western blotting was performed as described previously (29). The primary antibodies against steroidogenic enzymes (anti-Cyp17a1 and -Cyp11b2) diluted at 1:1000. α-tubulin was used as a reference protein and its abundance was used to normalize protein sample loading. Densitometry was quantified and normalized using α-tubulin as a reference protein using the Fusion-CAPT software.

### Germ cell counting

The cell count of spermatogenic cells at various stages (i.e. spermatogonia, primary spermatocyte and secondary spermatocyte) were calculated according to the methods described previously (31). Briefly, ten sections were randomly selected from each *cyp17a2* ^+/+^ (n=3) and *cyp17a2* ^-/-^ (n=3) XY fish at 90 dah, and the sections were stained with conventional hematoxylin and eosin (H&E) staining. Then the image of each section at 40x magnification was acquired with an Olympus BX5 light microscope (Olympus, Japan) for germ cell counting.

### Measurement of serum steroid hormone levels *via* EIA assay and LC-MS/MS

Blood samples were collected from the caudal veins of *cyp17a2* ^+/+^ XY (n≥3) and *cyp17a2* ^-/-^ XY (n≥3) fish after anesthesia (250 mg/L, MS-222, Sigma, St Louis, USA) at 180 dah and kept at room temperature for 1 h. After centrifugation at 3000 rpm for 10 min, the supernatant was transferred to a clean centrifuge tube. The supernatant was then centrifuged at 12000 rpm for 10 min at 4 °C and frozen at -80 °C until use. Serum steroid levels were measured using the EIA (enzyme-linked immunosorbent assay) kits (Cayman, Michigan, USA) or detected by MetWare (http://www.metware.cn/) based on the AB Sciex QTRAP^®^ 6500 LC-MS/MS system (AB Sciex, Framingham, USA). The steroid information was listed in **Table 2**.

**Table 2:** Key resources for steroids metabolism analysis.

MW ID	Compound Name	CAS
MWS0782	Testosterone	58-22-0
MWS0431	Dihydrotestosterone	521-18-6
MWS0785	Androstenedione	63-05-8
MWS0521	Cortisol	50-23-7
MWS0958	11-Dehydrocorticosterone	72-23-1
MWS0523	Deoxycorticosterone	64-85-7
MWS0797	Cortisone	53-06-5
MWS0784	17β-Estradiol	50-28-2
MWS0485	Estrone	53-16-7
MWS2130	2-Hydroxy Estrone	362-06-1
MWS0964	17α-Hydroxypregnenolone	387-79-1
MWS0833	5alpha-Pregnane-3,20-dione	566-65-4
MWS0799	Progesterone	57-83-0
MWS0524	17α-Hydroxyprogesterone	68-96-2
MWS0834	Pregnanediol	80-92-2
MWS0941	24-Hydroxycholesterol	474-73-7
MWS0936	7α,25-Dihydroxycholesterol	64907-22-8
MWS0138	Cholesterol	57-88-5
MWS0723	7-Hydroxy-cholesten-3-one	3862-25-7
MWS0727	20α-Hydroxycholesterol	516-72-3
MWS0730	7-Ketocholesterol	566-28-9
MWS1082	β-Sitosterol	83-46-5
MWS-20-102	Pregnenolone sulfate sodium salt	1852-38-6
MWS-20-73	Dehydroepiandrsterone	651-48-9

### Sperm characteristics and fertility assessment

To detect the impacts of *cyp17a2* gene mutation on spermatogenesis and fertility of male tilapia, changes of sperm morphology, sperm motility, and fertility were compared between *cyp17a2* ^-/-^ and *cyp17a2* ^+/+^ XY fish at 300 dah. Papanicolaou staining was used to detect the morphology of sperms according to the method which were used in previous reports (25, 31). Sperm motility, VCL (curvilinear velocity), VSL (straight line velocity) and sperm quality were examined *via* computer-assisted sperm analysis using the Sperm Quality Analyzer software according to the manufacturer’s instructions (Zoneking Software, China). The fertility of *cyp17a2* ^+/+^ and *cyp17a2* ^-/-^ XY fish was assessed *via* artificial insemination. Eggs from wild-type XX female fish (n=3) were divided into six groups (approximately 300 eggs/group) and artificial insemination was performed using sperm obtained from the *cyp17a2* ^+/+^ and *cyp17a2* ^-/-^ XY fish. Moreover, the survival rate of embryos was calculated from 1-9 days after fertilization.

### Data analysis

All the data for qRT-PCR, Western blotting, cell count, LC-MS/MS and EIA assay are presented as the mean ± SD. And Student’s *t*-test was performed to determine the difference between the two groups. P<0.05 was considered statistically significant differences.

## Results

### Cellular localization of Cyp17a2 antibody in tilapia gonads and head-kidney

**Figure 1:**
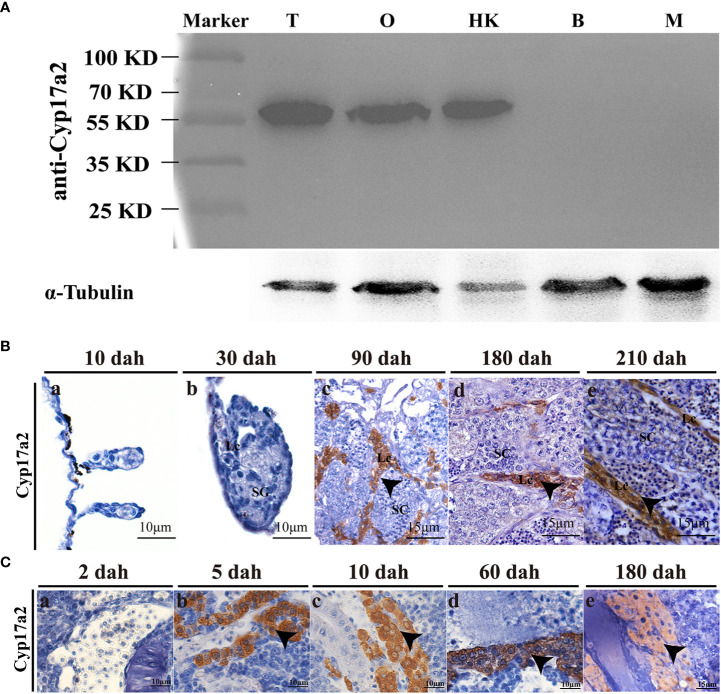
Expression profile of Cyp17a2 in tilapia. **(A)**, The specificity of polyclonal antibody against Cyp17a2 (~60 KD) in the testis, ovary, head-kidney, brain, and muscle was verified by Western blotting. T, testis; O, ovary; HK, head-kidney; B, brain; M, muscle. **(B)**, Cellular localization of Cyp17a2 in the testes was detected by IHC. Cyp17a2 was expressed in the Leydig cells of the testis at 90, 180, and 210 dah. SG, spermatogonia; SC, spermatocytes; Lc, Leydig cells. **(C)**, Cyp17a2 expression in the head-kidneys was detected by IHC. Cyp17a2 positive signals were detected in the interrenal cells of the head-kidney at 5, 10, 60, and 180 dah. Arrowhead, positive signals of Cyp17a2.

Total proteins extracted from gonads (ovary and testis), head-kidney, brain, and muscle were used to check the specificity of the Cyp17a2 polyclonal antibody by Western blotting. Western blotting showed that a single and specific band could be recognized in testis, ovary, and head-kidney, but not in brain and muscle by using tilapia Cyp17a2 antibody (**Figure 1A**). By IHC, positive Cyp17a2 immunostaining signals were observed in the Leydig cells of the testes in XY fish at 90, 180 and 210 dah (**Figure 1B**). In the head-kidney, Cyp17a2 was predominantly expressed in interrenal cells from 5 to 180 dah (**Figure 1C**).

### Generation of *cyp17a2* homozygous mutation line

**Figure 2:**
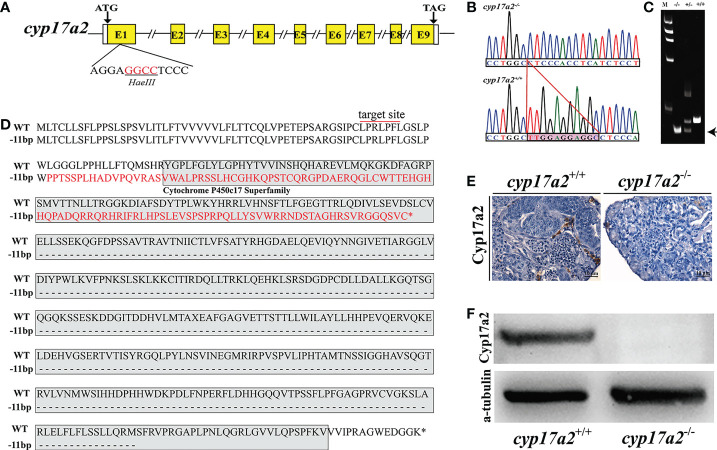
Establishment of *cyp17a2*-null mutation line by CRISPR/Cas9. **(A)**, Gene structure of *cyp17a2* gene with the target site and the underlined restriction enzyme (*Hae* III) cutting site. **(B)**, A deletion of 11 bp (TTGGAGGAGGC) in the genome of *cyp17a2* ^-/-^ fish compared with *cyp17a2* ^+/+^ fish was detected by Sanger sequencing analysis and the deletion region was highlighted by a pink shading. **(C)**, Mutations in the *cyp17a2* gene were detected by polyacrylamide gel electrophoresis. Wild type (+/+), heterozygous (+/-), and homozygous (-/-) fish were screened *via* PCR and PAGE gel electrophoresis. M, marker; Arrow, homoduplex of mutants **(D)**, A putative truncated protein was produced in *cyp17a2* ^-/-^ fish (-11 bp) compared with *cyp17a2* ^+/+^ fish (WT). Red line, target site; Red letters, putative amino acids in cyp17a2-/- fish; Gray shading, Cytochrome P450c17 Superfamily domain. Asterisk, termination of protein. **(E, F)** Cyp17a2 expression was not detected in the testes of *cyp17a2* ^-/-^ fish according to IHC and Western blotting analyses.

To investigate the potential roles of Cyp17a2 in fish steroidogenesis, a *cyp17a2* homozygous mutation line was produced using CRISPR/Cas9 technology by targeting the first exon (**Figure 2A**). A frame-shift mutation with 11-bp (TTGGAGGAGGC) deletion in *cyp17a2* homozygous mutant was obtained by Sanger sequencing (**Figure 2B**). A heteroduplex motility assay showed that heterozygous *cyp17a2* ^+/−^ individual was identified as possessing both heteroduplex and homoduplex amplicons, while the *cyp17a2* ^-/-^ and *cyp17a2* ^+/+^ individuals were found to be single band profile with only homoduplex amplicons (**Figure 2C**). The comparison of the predicted amino acid sequences demonstrated that the 11-bp deletion in the *cyp17a2* gene resulted in the production of truncated protein due to frame shift and the occurrence of a premature stop codon (**Figure 2D**). IHC and Western blotting analyses also demonstrated that Cyp17a2 protein completely vanished in the testes of *cyp17a2* ^-/-^ XY fish (**Figures 2E, F**).

### Decrease of cortisol production in *cyp17a2* ^-/-^ fish

**Figure 3:**
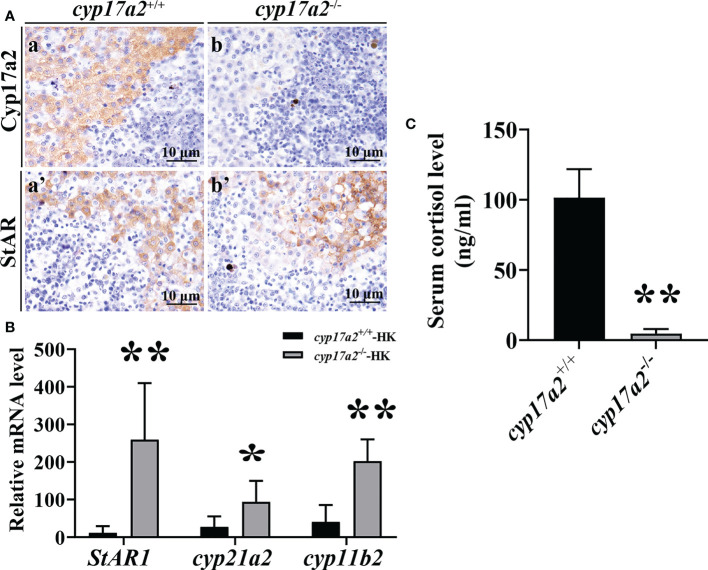
Effects of *cyp17a2* deficiency on steroid production in head-kidney of tilapia. **(A)**, Immunohistochemistry of Cyp17a2 (a and b) and StAR (a’ and b’) in the head-kidney of *cyp17a2* ^+/+^ and *cyp17a2* ^-/-^ XY tilapia. **(B)**, Expression level of *StAR1*, *cyp21a2*, and *cyp11b2* in the head-kidney of *cyp17a2* ^+/+^ (n=3) and *cyp17a2* ^-/-^ (n=3) fish detected by qRT-PCR. *β-actin* was used as a reference gene to normalize the expression values. **(C)**, Serum cortisol level in *cyp17a2* ^+/+^ (n=5) and *cyp17a2* ^-/-^ (n=5) fish measured using an EIA kit. The data are reported as the means ± SD. Asterisk above the error bar indicate significant differences between groups tested by Student’s *t*-test (*, P<0.05; **, P<0.01).

IHC results demonstrated that the expression of Cyp17a2 could not be detected in the head-kidney of *cyp17a2* ^-/-^ fish (**Figure 3A**). The results of qRT-PCR showed that the expression of *StAR1*, *cyp21a2* and *cyp11b2* in the head-kidney was significantly increased in *cyp17a2* ^-/-^ fish compared with wild-type fish (**Figure 3B**). EIA assay showed that serum cortisol level of *cyp17a2* ^-/-^ XY fish was significantly reduced (**Figure 3C**).

### Cyp17a2 is responsible for DHP, but not 11-KT biosynthesis

**Figure 4:**
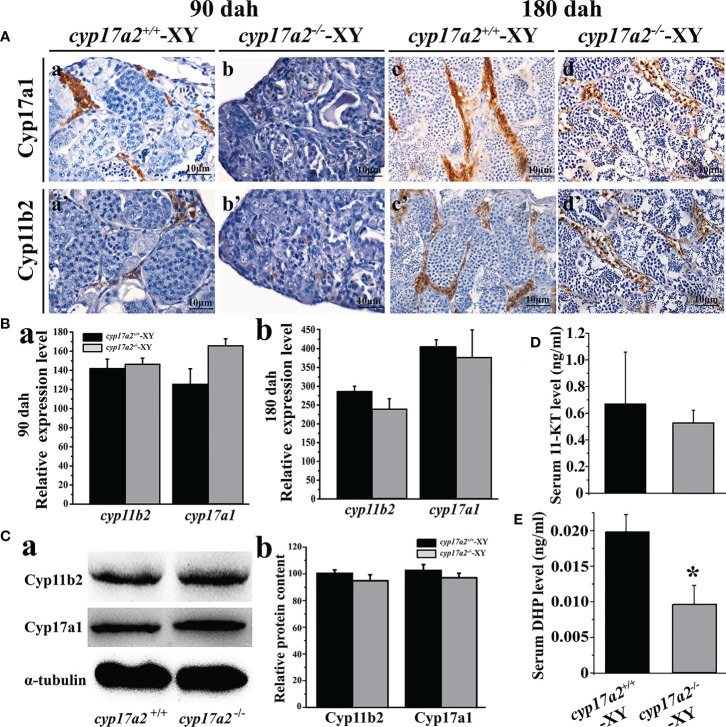
Effects of *cyp17a2* deficiency on steroid production in testis of tilapia. **(A)**, Immunohistochemistry of Cyp17a1 (a-d) and Cyp11b2 (a’-d’) in the testis of *cyp17a2* ^+/+^ and *cyp17a2* ^-/-^ XY tilapia at 90 and 180 dah. **(B)**, Expression level of *cyp11b2* and *cyp17a1* in the testis of *cyp17a2* ^+/+^ (n=3) and *cyp17a2* ^-/-^ (n=3) fish at 90 (a) and 180 dah (b) detected by qRT-PCR. *β-actin* was used as a reference gene to normalize the expression values. **(C)**, Expression of Cyp11b2 and Cyp17a1 analyzed by Western blotting (a) and quantification of protein level (b) in the testis of *cyp17a2* ^+/+^ and *cyp17a2* ^-/-^ at 180 dah. **(D, E)**, Serum 11-KT and DHP level in *cyp17a2* ^+/+^ (n=5) and *cyp17a2* ^-/-^ (n=5) fish measured using an EIA kit. The data are reported as the means ± SD. Asterisk above the error bar indicate significant differences between groups tested by Student’s *t*-test (*, P<0.05).

EIA assay showed that *cyp17a2* deficiency led to a significant decline in the biosynthesis of DHP, while no evident change of 11-KT production was detected between *cyp17a2* ^-/-^ and wild-type XY fish (**Figures 4D, E**). Moreover, IHC analysis demonstrated that the positive signals of steroidogenic enzymes (Cyp17a1 and Cyp11b2) concerning androgen production were detected both in the testes of *cyp17a2* ^-/-^ and wild-type XY fish (**Figure 4A**). Further analyses by qRT-PCR and Western blotting demonstrated that no significant difference in the expression level of androgen synthases (*cyp17a1*/Cyp17a1, *cyp11b2*/Cyp11b2) was found between *cyp17a2* ^-/-^ XY fish and wild-type XY fish at 90 and 180 dah (**Figures 4B, C**).

### The landscape of steroidogenesis in *cyp17a2* ^-/-^ male fish

**Figure 5:**
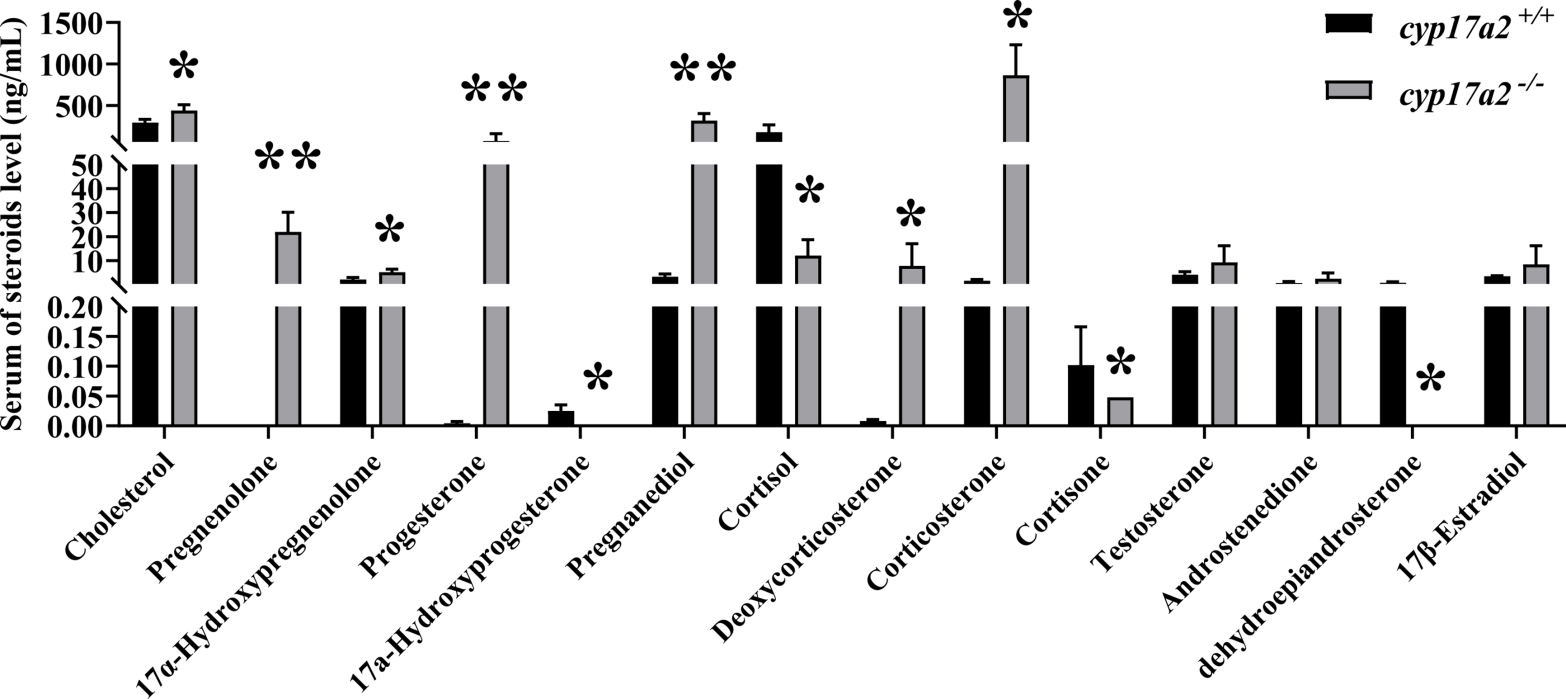
Mutation of *cyp17a2* resulted in disordered steroidogenesis in male tilapia. Serum steroid levels of *cyp17a2* ^+/+^ (n=3) and *cyp17a2* ^-/-^ (n=3) fish were measured by LC-MS/MS. The data are reported as the means ± SD. Asterisk above the error bar indicate significant differences between groups tested by Student’s *t*-test (*, P<0.05; **, P<0.01). .

Consistently, the results of LC-MS/MS showed that *cyp17a2* gene mutation led to significant changes in C21-steroid levels in *cyp17a2* ^-/-^ XY fish compared with wild-type XY fish. The syntheses of 17α-hydroxyprogesterone, cortisol and cortisone were significantly reduced in *cyp17a2* ^-/-^ XY fish, while cholesterol, pregnenolone, 17α-hydroxypregnenolone, progesterone, pregnanediol, deoxycorticosterone, and corticosterone were excessive accumulated (**Figure 5**). However, compared with wild-type XY fish, the production levels of C19-steroids (testosterone, androstenedione) and C18-steroid (17β-estradiol) were not affected (**Figure 5**).

### A deficiency of *cyp17a2* resulted in arrested meiotic initiation

**Figure 6:**
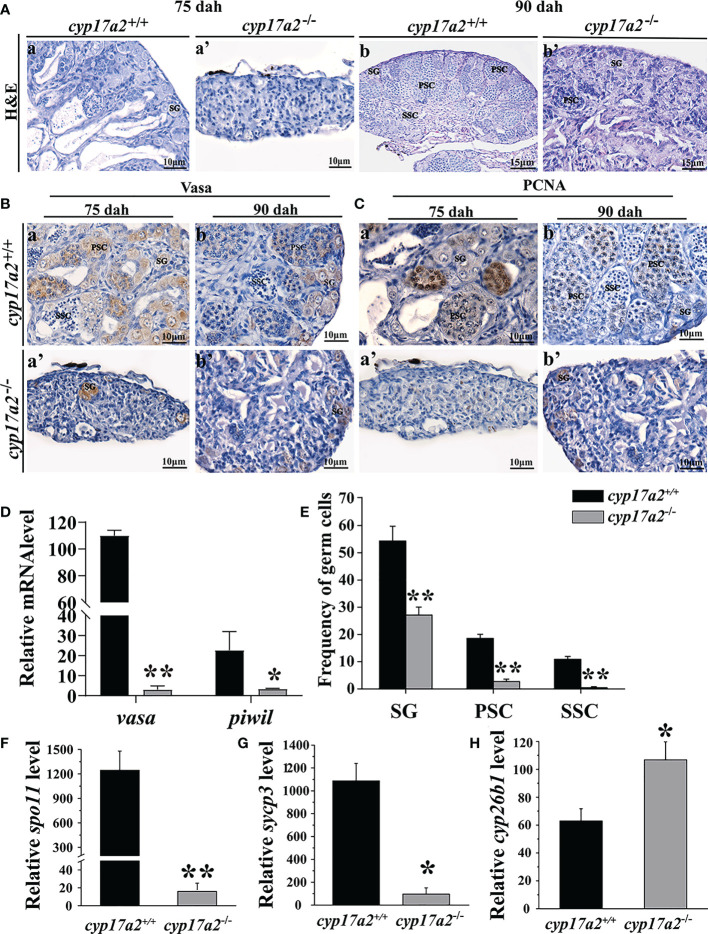
Mutation of *cyp17a2* resulted in delayed spermatogenesis. **(A)**, H&E staining of testis from *cyp17a2* ^+/+^ (a and b) and *cyp17a2* ^-/-^ XY fish (a’ and b’) at 75 and 90 dah. B and C, Immunohistochemistry of Vasa **(B)** and PCNA **(C)** in the testis of *cyp17a2* ^+/+^ and *cyp17a2* ^-/-^ XY fish at 75 and 90 dah. SG, spermatogonia; PSC, primary spermatocytes; SSC, secondary spermatocytes; ST, Spermatids. **(D)**, Expression level of *vasa* and *piwil* in the testis of *cyp17a2* ^+/+^ (n=3) and *cyp17a2* ^-/-^ (n=3) fish at 90 dah detected by qRT-PCR. *β-actin* was used as a reference gene to normalize the expression values. **(E)**, Spermatogenic cell proportion of *cyp17a2* ^+/+^ (n=3) and *cyp17a2* ^-/-^ (n=3) XY fish at 90 dah. **(F-H)**, Expression level of *spo11*, *sycp3*, and *cyp26b1* in the testis of *cyp17a2* ^+/+^ (n=3) and *cyp17a2* ^-/-^ (n=3) fish at 90 dah. *β-actin* was used as a reference gene to normalize the expression values. The data are reported as the means ± SD. Asterisk above the error bar indicate significant differences between groups tested by Student’s *t*-test (*, P<0.05; **, P<0.01).

To detect the role of Cyp17a2 in the initiation of meiosis in the development of tilapia testes, the gonad phenotype was evaluated at 75 and 90 days, respectively. This period was proved to be the key period for the initiation of meiosis and the morphological differentiation of gonads in wild-type male tilapia. H&E staining reflected that only a few spermatogonia and few spermatocytes were found in *cyp17a2* ^-/-^ XY fish compared with *cyp17a2* ^+/+^ XY fish at 75 and 90 dah (**Figure 6A**). Immunostaining showed that the number of Vasa (germ cell) and PCNA (proliferative cell) positive cells was significantly reduced in *cyp17a2* ^-/-^ XY fish than that in wild-type XY fish at 75 and 90 dah (**Figures 6B, C**). And the relative expression level of *vasa* and *piwil* was significantly decreased in *cyp17a2* ^-/-^ XY fish at 90 dah (**Figure 6D**). Consistently, a significant reduction of spermatogonia, primary spermatocytes, and secondary spermatocytes was detected in *cyp17a2* ^-/-^ XY fish at 90 dah (**Figure 6E**). In addition, qRT-PCR demonstrated that compared with *cyp17a2* ^+/+^ XY fish, the expression of *spo11*, *sycp3* (meiotic marker genes) were decreased while *cyp26b1* (retinoic acid hydrolase) was increased significantly in *cyp17a2* ^-/-^ XY fish at 90 dah (**Figures 6F-H**).

### Deficiency of Cyp17a2 resulted in subfertility in male tilapia

**Figure 7:**
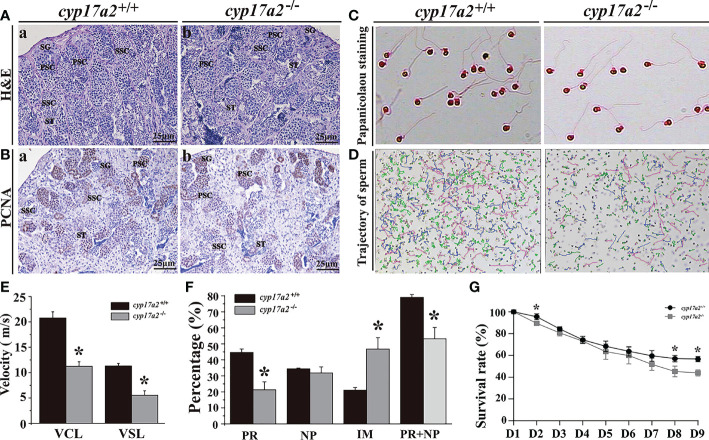
Mutation of *cyp17a2* gene reduced sperm quality. **(A)**, H&E staining of testis from *cyp17a2* ^+/+^ (a) and *cyp17a2* ^-/-^ (b) XY fish at 180 dah. **(B)**, Immunohistochemistry of PCNA in the testis of *cyp17a2* ^+/+^ (a) and *cyp17a2* ^-/-^ (b) XY fish at 180 dah. SG, spermatogonia; PSC, primary spermatocytes; SSC, secondary spermatocytes; ST, spermatids. **(C)**, Morphology of sperm from *cyp17a2* ^+/+^ (a) and *cyp17a2* ^-/-^ (b) XY fish. **(D)**, Trajectory of motile sperm from *cyp17a2* ^+/+^ (a) and *cyp17a2* ^-/-^ XY (b) fish. **(E)**, Average speed of VCL (curve movement speed) and VSL (straight line movement speed) in *cyp17a2* ^+/+^ and *cyp17a2* ^-/-^ XY fish. **(F)**, Quantification of different tracking parameters of PR (progressive sperm), PR+NP (non-progressive sperm) and IM (immotile sperm) in *cyp17a2* ^+/+^ and *cyp17a2* ^-/-^ XY fish. **(G)**, Survival rates of embryos that *cyp17a2* ^-/-^ and *cyp17a2* ^+/+^ XY fish artificially inseminate with normal females (n=3). D, Days after fertilization. Data are expressed as the mean ± SD. Asterisk above the error bar indicate significant differences between groups tested by Student’s *t*-test (*, P<0.05).

Surprisingly, spermatogenesis was restored at 180 dah, as indicated by the existence of all stages of spermatogenic cells and normal proliferation (**Figures 7A, B**). Papanicolaou staining showed that sperm from *cyp17a2* ^-/-^ XY fish showed normal sperm morphology (**Figure 7C**). However, low sperm motility was indicated by the trajectory and sperm tracking parameters (VCL and VSL) (**Figures 7D, E**). Quantification of the three sperm grades showed that dramatic attenuation of PR (progressive sperm) and NP (non-progressive sperm), while a significant increase in the frequency of IM (immotile sperm) was found in *cyp17a2* ^-/-^ XY fish compared to their control counterparts (**Figure 7F**). The fertilization rate and survival rate (D8 and D9) of *cyp17a2* ^-/-^ XY fish were significantly lower than those of *cyp17a2* ^+/+^ XY fish (**Figure 7G**).

## Discussion

Previous studies revealed that steroid hormones, i.e. 11-KT, DHP and cortisol, might be involved in spermatogenesis and male fertility in fish (8, 16, 32). Due to the fish-specific genome duplication, duplicated copies of several steroidogenic enzymes (two *StAR*, two *hsd3b*, two *cyp17*, two *cyp19a*) had been identified in fish genomes (20, 21, 33–36). Therefore, clarification of the distinct roles of the duplicated steroidogenic enzymes will be helpful to understand the intricacy of fish steroidogenesis. In teleosts, two paralogous *cyp17a* genes, named *cyp17a1* and *cyp17a2*, have been characterized (20–22). In this study, the homozygous mutation line of the *cyp17a2* gene was constructed in tilapia. We found that a deficiency of Cyp17a2 led to the sharp decline of DHP and cortisol, which further impaired sperm motility and male fertility.

Steroidogenesis is regulated sequentially by a series of steroidogenic enzymes and co-factors (37). In mammalian species, a single Cyp17a was responsible for the catalyzation of the biosynthesis of C18, C19 and C21 steroids in both gonads and the adrenal gland (18, 38, 39). In contrast, it was documented that Cyp17a1 and 2 might be involved in the biosynthesis of androgen and estrogen in gonads, and glucocorticoids in head-kidney of fish (20). Previous reports revealed that both Cyp17a1 and 2 demonstrates the different spatiotemporal expression profile and different enzymatic activities. *ISH* (*In situ* hybridization) experiments showed that two *cyp17a* genes were colocalized in the granulosa cells in the ovaries, and Leydig cells in the testis, while *cyp17a2* was specifically expressed in the interrenal cells in head-kidney in fish (20). In this study, an antibody against tilapia *cyp17a2* gene was produced, and expression of Cyp17a2 in Leydig cells and interrenal cells of male fish were further evinced. Previous reports by *in vitro* assay revealed that Cyp17a1, with both of the 17α-hydroxylase and 17, 20 layse activities, was capable of converting from progesterone and pregnenolone to androstenedione and dehydroepiandrosterone (DHEA) through 17α-hydroxyprogesterone and 17α-hydroxypregnenolone. On the contrary, Cyp17a2, with only 17α-hydroxylase activity, was able to catalyze the biosynthesis from pregnenolone and progesterone to 17α-hydroxypregnenolone and 17α-hydroxyprogesterone, respectively (20). The different expression profiles and distinct enzymatic activities suggested that Cyp17a1 and Cyp17a2 might be involved in different steroidogenic pathway. Therefore, it is intriguing to explore the functions of Cyp17a1 and 2 in fish by loss of gene functions. In tilapia, zebrafish (*Danio rerio*) and common carp (*Cyprinus carpio*), mutation of *cyp17a1* gene resulted in the blockage of androgen and estrogen synthesis, followed by sex reversal from female to male, suggesting that Cyp17a1 is essential for androgen and estrogen production in teleosts (23–25). On the contrary, we found that *cyp17a2* gene mutation had no effects on the production of both androgens and estrogens. Significant declines of both DHP and cortisol were detected in *cyp17a2* ^-/-^ XY fish indicated that *cyp17a2* was indispensable for these two steroids production in fish. In the head-kidney of *cyp17a2* ^-/-^ fish, an evident decrease of cortisol and striking up-regulation of *StAR1*, *cyp21a2* and *cyp11b2* was detected suggesting the critical role of Cyp17a2 in cortisol production. Taken together, functional analysis strongly emphasized that Cyp17a1 and 2 play distinct roles in androgen, estrogen and glucocorticoids production in fish. In the testis, Cyp17a1 was involved in the production of 11-KT in Leydig cells, which is required for both spermatogenesis and spermiation (25). Our present study by *cyp17a2* gene editing further proved that the expression of Cyp17a2 in Leydig cells was responsible for DHP production in XY fish. Undoubtedly, this study has proved that specific expression of *cyp17a2* in the interrenal cells of head-kidney was responsible for cortisol production in fish.

In fish, the production of 11-KT and DHP in Leydig cells in the testis were strictly controlled by gonadotropins from pituitary (40). In our present study, a significant decline in DHP biosynthesis was detected in serum of *cyp17a2* ^-/-^ tilapia, indicating that Cyp17a2 is required for DHP production in male fish. However, a low DHP level was still detected in *cyp17a2* ^-/-^ XY, so it is not excluded that the residual DHP production might be catalyzed by other enzymes *via* genetic compensation response in *cyp17a2* ^-/-^ mutants. And the decline in DHP production did not affect 11-KT production. Furthermore, the stable expression of downstream steroidogenic genes (*cyp17a1*/Cyp17a1 and *cyp11b2*/Cyp11b2) was found in the testis of *cyp17a2* ^-/-^ XY fish. These findings indicated the independence of DHP and 11-KT production in the testicular Leydig cells of tilapia. Consistently, our previous work found that RU486 treatment, an antagonist of DHP, had no effects on the biosynthesis of 11-KT and the expression of steroidogenic enzymes for 11-KT production (41).

For a long time, the involvements of 11-KT in spermatogenesis and testis development have been extensively studied by short-term culture experiments and gene editing tools (32, 42–44). Recent studies revealed that DHP and cortisol might also play essential roles in testicular differentiation. It was documented that cortisol treatment induced DNA replication in spermatogonia, and further enhanced 11-KT-induced spermatogonial proliferation (16). In fish, DHP had been proved to be a maturation-inducing hormone and it was increased sharply during oocytes maturation and ovulation (45). In Japanese ell (*Anguilla japonica*), using an *in vitro* testicular culture system, DHP was shown to induce DNA replication in spermatogonia (8). Furthermore, DHP treatment also induced the expression of meiosis-specific markers, such as Dmc1 and Spo11 highlighting their essentiality in meiotic initiation (8). Our previous work found that blockage of the DHP-Pgr signaling pathway by RU486 treatment led to the retardation of spermatogenesis and sperm maturation indicating the essential role of DHP production in male spermatogenesis and fertility (41). In fish, previous reports demonstrated Cyp17a2 might be one of the key enzymes to promote the production of both DHP and cortisol based on its expression profiles and enzymatic activities (20). In the present study, we found that *cyp17a2* deficiency led to the significant decline of both DHP and cortisol, delay of meiotic initiation during the early stage of testicular differentiation revealing the decreased expression of meiosis related genes (*spo11* and *sycp3*), and increased expression of retinoic acid hydrolase (*cyp26b1*). These findings indicated that DHP and cortisol production might be indispensable for the initiation of spermatogenesis in male tilapia.

It was also well documented that DHP played crucial roles in final sperm maturation, spermiation, and sperm motility (10). In male salmonid fish, a peak of DHP was found in blood plasma in the spawning season (46). In turbot (*Scophthalmus maximus*), males with higher sperm motility showed higher levels of DHP compared with males with lower sperm motility, which indicated that DHP might induce spermatogenesis and regulate sperm motility (47). Reports showed that DHP might be involved in seminal fluid production, spermatogonial proliferation and spermatogenesis (9, 48, 49). Knockout of the progesterone receptor gene, *pgr*, led to the decline of sperm count and sperm motility in male tilapia, indicating the importance of the DHP-Pgr signaling pathway in fish sperm maturation (31). Consistently, deficiency of c*yp17a2* led to the decline of DHP and cortisol production, which resulted in the decline of sperm motility and subfertility in male tilapia. Through functional study, our present study further proved that DHP and cortisol played an essential role in spermiogenesis and fertility in male fish. In zebrafish, mutation of *cyp17a2* gene resulted in the enlargement of interrenal gland, up-regulated expression of *cyp11a1*, *cyp21a1*, and *hsd3b1* in the interrenal gland, and significant decrease of cortisol biosynthesis (50). However, zebrafish *cyp17a2* gene mutation had no effects on fertility and secondary sex characteristics in males. Furthermore, abnormal increase of progesterone, testosterone, DHP, and 11-KT in the *cyp17a2*-deficient males than those in wild-type male fish was observed (50). We speculated that discrepancy of DHP production and male fertility between *cyp17a2* deficiency zebrafish and tilapia might be due to the species difference.

Taken together, our findings, by using a homozygous mutation line of *cyp17a2* gene, proved that Cyp17a2 was involved in the biosynthesis of DHP and cortisol in tilapia, which were essential for meiotic initiation, spermiogenesis and male fertility.

## Data availability statement

The datasets presented in this study can be found in online repositories. The names of the repository/repositories and accession number(s) can be found in the article/supplementary material.

## Ethics statement

The animal study was reviewed and approved by Institutional Animal Care and Use Committee of Southwest University.

## Author contributions

LZ and LY conceived and designed the experiments; LY and YW performed most of the experiments. YS and XZ maintained the fish stocks. LZ and LY analyzed the data, interpreted the results, and drafted the manuscript; LZ, DW, and TC edited the manuscript. All authors read and approved the final version of this manuscript.

## Funding

This work was supported by the National Natural Science Foundation of China (32072957, 31772825), Natural Science Foundation of Chongqing (cst2021jcjy-msxm0088), Scientific Research Innovation Project for Chongqing Postgraduates (CYS21105).

## Conflict of interest

The authors declare that the research was conducted in the absence of any commercial or financial relationships that could be construed as a potential conflict of interest.

## Publisher’s note

All claims expressed in this article are solely those of the authors and do not necessarily represent those of their affiliated organizations, or those of the publisher, the editors and the reviewers. Any product that may be evaluated in this article, or claim that may be made by its manufacturer, is not guaranteed or endorsed by the publisher.

## Abbreviations

11-KT, 11-ketotestosterone; Cyp11b2, Cytochrome P450, family 11, subfamily B, polypeptide 2; Cyp17a1, Cytochrome P450 family 17 subfamily A member 1; DHP, 17α, 20β-dihydroxy-4-pregnen-3-one.
